# Robust Localization for Underground Mining Vehicles: An Application in a Room and Pillar Mine

**DOI:** 10.3390/s23198059

**Published:** 2023-09-24

**Authors:** Felipe Inostroza, Isao Parra-Tsunekawa, Javier Ruiz-del-Solar

**Affiliations:** 1 Advanced Mining Technology Center, Universidad de Chile, Santiago 8370451, Chile; felipe.inostroza@amtc.uchile.cl (F.I.); isao.parra@amtc.uchile.cl (I.P.-T.); 2 Department of Electrical Engineering, Universidad de Chile, Santiago 8370451, Chile

**Keywords:** localization in underground mines, SLAM, autonomous mining, autonomous load-haul-dump, room & pillar mines

## Abstract

Most autonomous navigation systems used in underground mining vehicles such as load–haul–dump (LHD) vehicles and trucks use 2D light detection and ranging (LIDAR) sensors and 2D representations/maps of the environment. In this article, we propose the use of 3D LIDARs and existing 3D simultaneous localization and mapping (SLAM) jointly with 2D mapping methods to produce or update 2D grid maps of underground tunnels that may have significant elevation changes. Existing mapping methods that only use 2D LIDARs are shown to fail to produce accurate 2D grid maps of the environment. These maps can be used for robust localization and navigation in different mine types (e.g., sublevel stoping, block/panel caving, room and pillar), using only 2D LIDAR sensors. The proposed methodology was tested in the Werra Potash Mine located at Philippsthal, Germany, under real operational conditions. The obtained results show that the enhanced 2D map-building method produces a superior mapping performance compared with a 2D map generated without the use of the 3D LIDAR-based mapping solution. The 2D map generated enables robust 2D localization, which was tested during the operation of an autonomous LHD, performing autonomous navigation and autonomous loading over extended periods of time.

## 1. Introduction

**Figure 1:**
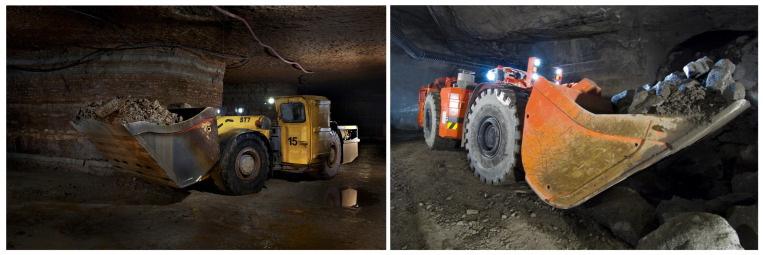
Examples images of LHD vehicles. **Left** image [3], **right** image [4].

The use of automated equipment is crucial in today’s mining industry. Autonomous vehicles allow for an increase in the safety, productivity, and efficiency of operations. In the case of underground mining, special care has been given to the automation of load–haul–dump (LHD) vehicles because they operate in high-risk areas [1], but also because the mine production directly depends on the operation of these vehicles, which move the material along the production areas. Moreover, the same technology used for automating LHD vehicles has been applied to the automation of low-profile trucks operating inside underground mines. LHD vehicles, example images of which are shown in Figure 1, are articulated industrial mining vehicles of considerable size, with lengths starting from 7 m up to more than 12 and weights of 16–41 tonnes plus payloads that range between 3.5 and 21 tonnes [2]. The weight, size, and kinematics of LHD vehicles plus the fact that they are hydraulic machines make the automation of these vehicles a significant challenge.

Commercial systems that automate the operation of LHD vehicles already exist (e.g., [5,6]). These systems are based on the use of 2D light detection and rangings (LIDARs) as their main sensors [6,7], and basically automate the hauling and dumping tasks. The loading of material is still implemented via tele-operation from different mine areas, or from outside the mine. However, there exist some new autonomous loading systems whose technological maturity will allow their commercial use in the next few years [8]. The operation of autonomous LHD vehicles requires that they move autonomously to the draw points and to the dumping points. These operations are executed repeatedly in each shift.

The navigation of LHD vehicles inside mining tunnels has very different requirements to the navigation of traditional robots inside mining tunnels (e.g., drones or mobile platforms exploring a mine). In the case of LHD vehicles, the main requirements are navigation at a high speed, in order to obtain a short operation cycle, and robust operation, i.e., being able to avoid any possible collision and never stopping during the shift. On the other hand, being able to navigate completely unexplored areas is typically not a requirement.

Autonomous LHD vehicles operate inside a so-called autonomous operation zone (AOZ). This corresponds to the area where the vehicle is expected to operate autonomously but segregated from human-operated vehicles. Redundant confinement methods are used in order to prevent any autonomous movement of the LHD outside the AOZ, as well as the entry of humans to the AOZ [9].

One of the critical factors that prevents autonomous mining vehicles achieving high speed and robustness at the same time, while moving along the tunnels of a production area, is the localization method employed for determining their own pose (position and orientation) at any given moment. The localization performance depends on the sensors and maps (mine representation) being used, which are built during the commissioning period. However, the performance also depends on the mining method (block caving, sublevel stoping, room and pillar, etc.). Thus, localizing an LHD inside a room and pillar mine is challenging because of the self-similarity/repeating pattern of the layout (room and pillar tunnels and intersections look resemble each other), the changes in tunnel elevation of up to several meters inside the AOZ, and the fact that the area where the material is loaded continuously expands due to blasting, which requires re-mapping during operation.

To the best of the author’s knowledge, there are no autonomous LHD vehicle developments operating in room and pillar mines in the world.

This article addresses these issues by proposing the joint use of 2D and 3D LIDARs for building consistent and accurate 2D maps used for representing the mine, which enable the accurate and reliable self-localization capability of autonomous LHD vehicles. The proposed methodology was validated inside the production area of a room and pillar mine using a real size LHD, which confirmed that the proposed approach can be used to overcome the localization challenges that occur in room and pillar mines.

The main contributions of this paper are:

- The combined use of 2D and 3D LIDARs and simultaneous localization and mapping (SLAM) algorithms to produce consistent 2D grid maps of underground tunnels that are only approximately 2D, which can be used in industrial applications, such as the one of LHD machines operating autonomously in the production tunnels of room and pillar mines.
- A heuristic method to produce incremental map updates with minimal human intervention, which is suited to be used in real mining applications where blasting is continuously used to expand the operation area of the mine, without interrupting the autonomous operation of the LHD vehicles.
- An industrial validation of the proposed 2D map building and localization systems, both in a real room and pillar mining environment, where the flat world assumption is partially broken, and using real mining equipment (an LHD) during autonomous operation, including muck pile loading, hauling, and dumping the loaded material.

## 2. Background and Literature Review

### 2.1. Mapping and Localization

A robot, or any autonomous vehicle, needs a map of its environment in order to work autonomously. The map creation process requires that the robot is localized while building the map. For this reason, this process is referred to as SLAM (due to the high correlation between map and localization, these problems need to be solved simultaneously [10]), and it has been one of the principal research areas in robotics ever since the field started [10].

A 2D SLAM algorithm builds a 2D map of the environment and localizes the robot in this map, while a 3D SLAM algorithm performs the equivalent task with a 3D map and 3D localization. The 2D SLAM problem has been extensively researched and solutions exist using Kalman filters [11], particle filters [12], global bundle adjustment/sparse optimization [13,14], etc. Examples of publicly available SLAM implementations using 2D LIDARs include SLAM Gmapping [15], based on the Rao-Blackwellized Particle Filter, and Google Cartographer [16], based on bundle adjustment. On the other hand, the 3D SLAM problem is an active area of research with optimization/bundle adjustment approaches typically outperforming filter-based solutions [17]. Examples of 3D LIDAR-based mapping algorithms include LIDAR odometry and mapping (LOAM) [18], and continous time-iterative closest point (ICP) (CT-ICP) [19]. Publicly available solutions include lightweight and ground optimized LOAM (LeGo-LOAM) [20] and LIO-SAM [21]. Recent advances on SLAM in underground environments are being published based on the DARPA Subterranean Challenge [22] datasets and results, such as LAMP [23] LAMP2.0 [24], LOCUS [25], and LOCUS2.0 [26]. Koval et al. [27] evaluated multiple 3D SLAM algorithms using data from the Darpa subt Challenge, namely BLAM [28,29], LOAM [18], A-LOAM [30], ISC-LOAM [31], LeGo-LOAM [20], LIO-mapping [32], Fast-LIO [33], F-LOAM [34], hdl_graph_slam [35], Cartographer [16], and LIO-SAM [21]. Results in the underground dataset showed varying performances, LeGo-LOAM being among the best-performing algorithms. Since results were published in 2022, while the testing of the system described in this article started in October 2021, this evaluation was therefore not considered for the algorithm selection..

The localization problem, due to its lower dimensionality (typically three dimensions for SE2 or six for SE3; since SE2 and SE3 are the special Euclidean groups in 2D and 3D spaces, respectively, these groups correspond to the space of 2D and 3D poses, including position and orientation), contrary to the typically thousands of dimensions of the SLAM problem, allows for lower complexity and/or higher robustness. This occurs due to the fact that state estimation suffers from the ‘*curse of dimensionality*’, where the difficulty of the problem increases super-linearly with the number of dimensions of the state being estimated [36].

Examples of existing 2D LIDAR localization algorithms in the robotics literature include Monte Carlo approaches, such as Monte Carlo localization (MCL) [10], adaptive Monte Carlo localization (AMCL) [37], and scan matching approaches, like normal distribution transform (NDT) [38]. Three-dimensional localization examples include direct LIDAR localization (DLL) [39].

In mining, published localization approaches to autonomous underground mining vehicles mainly use 2D LIDAR for localizing the vehicle [7,40,41,42,43], due to the robustness of these sensors and their operation range. For instance, in [40], Mäkelä combines 2D LIDAR data with odometry and a single-axis gyro through the use of a Kalman Filter. Scheding et al. uses an improved motion model for the articulated vehicle along with a bearing-only sensor that detects retro-reflectors [44]. Bakambu and Polotski reported a system that localized using tunnel-specific landmarks. However, this work was carried out prior to the existence of any real-time SLAM solutions [45]. Stefaniak et al. created a rough localization method based on extremely low-cost sensors (speed and a three-axis accelerometer) with comparable performance to RFID sensors that are used in underground mines [46]. In more recent work, Nielsen and Hendeby used 2D LIDAR descriptors applied to SLAM in underground mines. These descriptors were processed in a multi-hypothesis approach and applied using extended Kalman filter (EKF)-SLAM (EKF-SLAM); however, the approach should be applicable using other SLAM algorithms [43]. Further details of this approach can be found in [47]. Li et al. proposed an ultra wide band (UWB) sensor system to localize a vehicle in an underground coal mine using beacons [48]. Currently, the robust use of 3D LIDARs in mining vehicles is actively being developed, following the lead of the autonomous vehicles designed for public roads. Li et al. shows a real-time SLAM system that compensates for the degeneracy in the 3D LIDAR data produced in highly structured tunnels by using inertial measurement unit (IMU) data [48]. Ren and Wang proposed a 3D LIDAR-based localization system using an unscented Kalman filter (UKF) and a distance weight map (DWB) [49]. Tabib and Michael used a Gaussian mixture model to perform 3D SLAM in a cave of about 10 by 40 m [50].

Two-dimensional mapping and localization approaches such as the ones described previously do not typically address the uneven ground situation present in the room and pillar where these tests were conducted, while 3D LIDAR map-building systems typically require a 3D sensor for localization as well. Most published 2D SLAM and localization work focuses either on indoor scenarios [15,51], where the flat world assumption is very accurate, or on outdoor autonomous vehicle applications, in which the flat world assumption is partially violated [52,53]. The underground mining scenario presents the combined challenge of both these situations where the flat world assumption is partially violated, but due to it still being *indoors*, the 2D LIDAR data is occluded not only by the tunnel floor but also the ceiling. Additionally, the requirements of an industrial application, mainly high reliability, increase the difficulty of achieving a successful solution.

### 2.2. Mapping and Localization in Room and Pillar Mines

The proposed mapping and localization methodology is designed to be used by autonomous vehicles (e.g., LHD and trucks) operating in Room and Pillar mines. The proposed methodology is intended to be used in conjunction with the autonomous navigation system published by Mascaró et al. [41], which was originally designed to be used in block/panel caving mines and in sublevel stoping mines. This system localizes and navigates entirely topologically by following the tunnel structure of the mine. However, in room and pillar mines, robust metric maps and localization algorithms are required for the deployment of desirable features, such as forbidden zones, virtual obstacles (static objects to be avoided, which are not detected by the vehicle’s sensors), and more precise target poses for navigation (e.g., the material drop point). Therefore, in the process of adapting the autonomous navigation system to room and pillar type mining environments, a new metric map building and localization subsystem was developed. Keeping the existing topological map structure allows the system to efficiently and optimally solve the path planning problem in the tunnel graph representation.

The main requirements of the localization functionality are safe operation while maximizing speed to maximize mine productivity. However, occasional remote human intervention is permissible, and the initial setup can also include human intervention. This means that human intervention is appropriate during map building and that the localization module can be also initialized by human operators. Nevertheless, the localization module should be fast, reliable, and computationally inexpensive. Also, given that the autonomous navigation system is 2D-based, any pose will be converted into 2D and evaluated by examining the autonomous system performance.

Given these requirements and available sensors (2D and 3D LIDAR), there are multiple approaches to both the self-localization and map-building problems. Table 1 shows the possible approaches to map building. Rows indicate the type of map being built, with 3D maps including the height (z axis) information in their map, while 2D maps assume a constant height, producing a map that assumes a “flat world”. Columns indicate the type of LIDAR sensor used for sensing during map building. The table cells show examples of state-of-the-art algorithms using these approaches. All these algorithms are either available as ROS packages, or are easily implemented by adapting a source code available online. As can be seen from the table, the map can either be 3D or 2D, and the sensor used could be either 2D LIDAR or 3D LIDAR. Existing solutions to build a 3D map using 2D LIDARs [54] require both a vertically mounted 2D LIDAR and 2D LIDAR mounted on an inclined plane on a rotating platform. This setup is not practical in the case of mining vehicles operating in tunnels (e.g., LHD vehicles) because of the constraints on the vehicle’s places where the sensors can be mounted. Because of this and its cost-effectiveness compared to the currently available 3D LIDARs, this approach was discarded.

Table 2 shows examples of possible self-localization solutions. Rows indicate the type of map in which the vehicle will localize, produced by the map-building algorithm selected from Table 1. Columns indicate whether the localization will estimate a 3D pose (i.e., a pose in SE3) or a 2D pose (i.e., a pose in SE2). Note that methods to estimate a 3D pose using a 2D map are not known to the authors and therefore the corresponding cell is greyed out. If a 3D pose is estimated, the pose needs to be converted into a 2D pose for its use in navigation. The direct estimation of a 2D pose requires an assumption about the extra degrees of freedom of the 3D pose (height, roll, and pitch), possibly assuming constant values. The 2D map/2D localization approach was chosen in this document because of the reduced computational complexity, and the more mature availability of existing solutions. Note that the map-building process still makes use of the 3D LIDAR.

## 3. Materials and Methods

### 3.1. Methodology

This paper proposes to use 3D LIDAR sensors for building the mine maps and 2D LIDAR sensors for the vehicles’ localization inside the map. A 3D SLAM solution generated by a state-of-the-art algorithm is adapted and used to build the 2D map of the mine: the SLAM process is carried out in 3D, and using the 3D trajectory produced by the 3D SLAM algorithm, a 2D mapping algorithm estimates the map, without estimating the trajectory of the vehicle. The resulting 3D-assisted 2D map is used for the localization of the vehicles using 2D LIDARs as sensors.

Therefore, the proposed methodology consists of three main steps: (i) 3D map building; (ii) 3D-to-2D map conversion, and (iii) 2D self-localization. Of these three steps, only the third one is required to run during vehicle operation, while the map building and 3D–2D conversion steps just need to be run during the initial setup. Additionally, a fourth step, *map update*, can be executed to increase or modify the AOZ (e.g., after several load operations, or after a blast). These four steps will be described in the following sections.

### 3.2. Three-Dimensional Map Building

**Figure 2:**
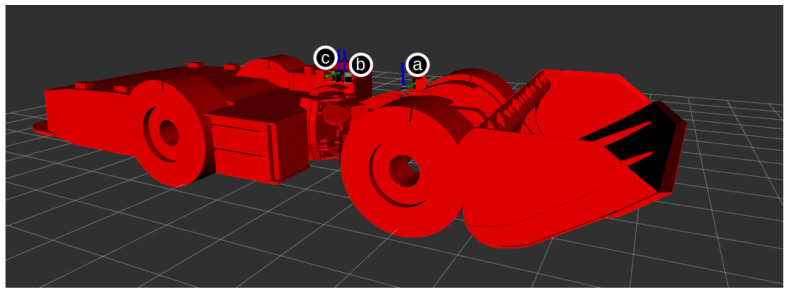
The ROS robot model of the vehicle, with LIDAR poses shown on top of the vehicle: (a) 3D LIDAR; (b) 2D LIDAR directed towards bucket side; and (c) 2D LIDAR directed towards the engine side.

In order to use the information provided by the 3D LIDAR, a 3D LIDAR-based SLAM algorithm needs to be chosen. To select a suitable algorithm to perform 3D SLAM prior to the field tests, a simulated room and pillar environment was assembled using environments built for the DARPA Subterranean Challenge [22] as building blocks. This new environment was then simulated using the Ignition Gazebo simulator, the same used in the DARPA Subterranean Challenge original environments. Using this simulator, a model of the LHD vehicle to be used in the real mine environment was created. The dimensions of the vehicle and sensor locations and resolutions were defined to be the same as those to be used during the mine tests reported in this work. Figure 2 shows the vehicle model used by the simulator. The kinematics of the vehicle were simulated; however, the hydraulics of the vehicle articulation and drive train were not. The simulated vehicle was teleoperated to go through the whole AOZ and back to the starting position, without sudden accelerations. Using this procedure, several 3D LIDAR-based SLAM algorithms were tested. The algorithms were selected based on their use of a 3D sensor and the availability of public code to execute them. The selected algorithms were LOAM [18], LeGo-LOAM [20], and Cartographer3D [16].

LeGo-LOAM [20], which is based on the LOAM [18] algorithm, works by exploiting the asymmetrical resolution of typical 3D LIDARs. This is because, since 3D LIDARs usually have a fixed number of horizontally rotating layers (typical number of layers are 10–64), each layer has high horizontal resolution (e.g., 0.25º). These algorithms extract features, corners, and planar sections from each layer. By calculating a curvature measure *c*, for each point in a row:

(1) $$c=\frac{1}{\left|S|\right|r_{i}|}∥\sum_{j\in S,j\neq i}\left(r_{j}-r_{i}\right)∥$$

In (1), the curvature at point *i* with range $r_{i}$ is calculated, and *S* is a set of continuous points, half on either side of *i*. Extracting the minimum and maximum from the curvature measure, *c* corners and planar sections can be extracted. Using these two sets of landmarks, an optimization approach is carried out to estimate the pose of the vehicle during a single revolution of the LIDAR. Another lower frequency loop refines the odometric result by carrying out a batch optimization between the poses at the start of each scan. Using the LIDAR odometries as inputs, LeGo-LOAM modifies the LOAM algorithm by including the ground data in the optimization, which is discarded by the original LOAM, as well as including possible loop closures based on ICP scan matching [20].

**Figure 3:**
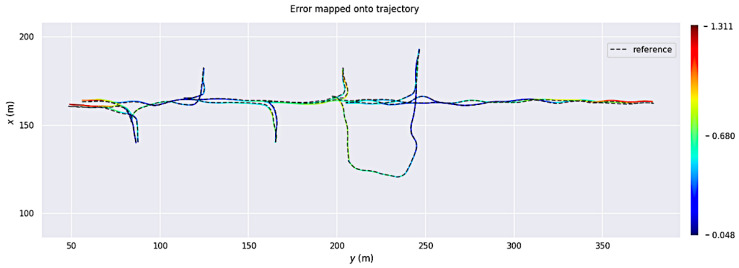
Absolute pose error of LOAM results on custom room and pillar simulation. The colored solid line shows the estimated trajectory with the color representing an instantaneous APE error. The ground truth trajectory is shown as a dashed gray line.

**Figure 4:**
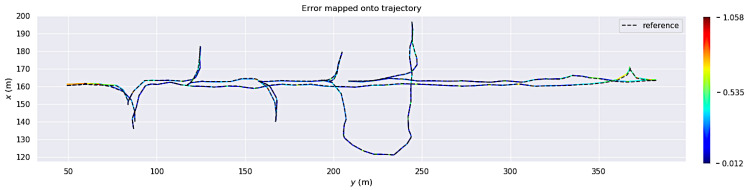
Absolute pose error of LeGo-LOAM results on custom room and pillar simulation. The colored solid line shows the estimated trajectory with the color representing an instantaneous APE error. Ground truth trajectory is shown as a dashed gray line.

**Figure 5:**
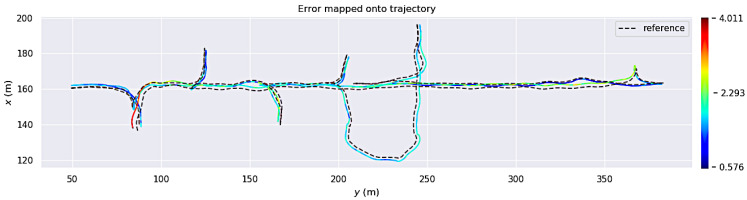
Absolute pose error of Cartographer3D results on custom room and pillar simulation. The colored solid line shows the estimated trajectory with the color representing instantaneous APE error. Ground truth trajectory is shown as a dashed gray line.

All three algorithms were evaluated based on their absolute pose error (APE) using the *evo* Python package [55]. Figure 3, Figure 4 and Figure 5 show the estimated trajectories and APE error metric for LOAM, LeGo-LOAM, and Cartographer3D, respectively. These three figures show that, in the custom simulated room and pillar environment, both LOAM and LeGo-LOAM perform satisfactorily, while Cartographer3D does not estimate the entire trajectory correctly. This could be explained by the fact that both LOAM and LeGo-LOAM are specifically designed for the type of 3D LIDAR available on the vehicle, which has a much higher horizontal resolution than vertical resolution, as described earlier in this section. Out of the first two algorithms, LeGo-LOAM was selected due to it having a BSD open source software license [20], which allows any use, including commercial. Additionally, LeGo-LOAM’s use of ground landmarks should theoretically help in the tunnel environment where there are not many landmarks (neither on the ground nor on the walls), although this was not shown in the simulation results.

### 3.3. Three-Dimensional-to-Two-Dimensional Conversion

To convert the 3D SLAM results into a 2D map, the simplest way is to take a slice of the 3D map at a single height. However, unless the floor is completely level, as in indoor scenarios, the single height will not produce a consistent 2D map. In the autonomous operation zone, where the mining vehicle has to operate, the tunnel elevation may change. In the case of a room and pillar mine, changes can be up to several meters. Therefore, assuming a constant height for map conversion is not possible.

To produce a consistent 2D map assisted by the 3D SLAM results, a mapping algorithm is required. In the robotics literature, mapping is distinguished from SLAM in that mapping uses a known vehicle trajectory to only estimate the map, while SLAM estimates both the map and the trajectory. Mapping is equivalent to using a state-of-the-art 2D SLAM algorithm with zero odometry noise. Due to its availability and known performance, the ROS package GMapping [16] is used for building the map.

The GMapping algorithm is fed with data generated by two 2D LIDARs mounted horizontally on the vehicle, one pointing forwards and one pointing backward. The 2D SLAM algorithm uses the 3D trajectory generated by the 3D algorithm, LeGo-LOAM, as odometry. To do this, the 3D trajectory has to be converted into a 2D one, i.e., each pose in the trajectory needs to have its position and orientation converted from 3D to 2D. As the 3D trajectory generated by 3D SLAM is approximately 2D, this can be performed by setting the pose’s z coordinate (height) value to 0. Similarly, each 3D orientation is converted to 2D by setting the pose’s roll and pitch values to 0 and only using the pose’s yaw value, with an Euler angle representation. Using this converted trajectory as odometry input, and setting the odometry noise parameters to very low levels, GMapping is used to produce a 2D grid map, concluding the initial map-building procedure.

### 3.4. To-Dimensional Localization

With the consistent occupancy grid map resulting from the previous section, the state-of-the-art AMCL method is implemented, using the publicly available AMCL package [37], available as a ROS package. MCL algorithms work by implementing a particle filter (also referred to as sequential Monte Carlo) to estimate the posterior distribution of the vehicle pose at time *k*, $x_{k}$, given all odometry inputs up to time k−1, $u_{0:k-1}$ and all measurements up to time *k*, $z_{0:k}$:

(2) $$p(x_{k}|u_{0:k-1},z_{0:k})$$

To estimate this posterior, the prior at time k−1, $p(x_{k-1}|u_{0:k-2},z_{0:k-1})$ is approximated by a sum of Dirac’s delta distributions:

(3) $$p\left(x_{k-1}|u_{0:k-2},z_{0:k-1}\right)\approx \sum_{i=1}^{N}w_{i}^{k-1}\delta \left(x_{k-1}-\mu_{i}^{k-1}\right)$$

In (3), $w_{i}^{k-1}$ and $\mu_{i}^{k-1}$ are the weights and locations of each particle at time k−1. Then, by sampling $\mu_{i}^{k}$ from the motion model of the vehicle $p(x_{k}|x_{k-1}=\mu_{i}^{k-1},u_{k-1})$ and weighting the new particles of the according to the measurement model $w_{i}^{k}=w_{i}^{k-1}p\left(z_{k}|x_{k}=\mu_{i}^{k}\right)$, an approximation for the posterior pose distribution at time *k* is obtained:

(4) $$p\left(x_{k}|u_{0:k-1},z_{0:k}\right)\approx \sum_{i=1}^{N}w_{i}^{k}\delta \left(x_{k}-\mu_{i}^{k}\right)$$

AMCL improves on the MCL algorithm by dynamically selecting the number of particles *N* used to represent the vehicle pose distribution. This dynamic *N* is calculated to keep the Kullback–Liebler divergence between the approximated distribution and the true posterior distribution, i.e., the left and right sides of (4), below a certain threshold [10].

#### Localization While Loading

The localization of the LHD while loading needs to be carefully tuned. During loading, the vehicle pushes against the pile of material being loaded, significantly increasing the wheel slip. Therefore, when using the same odometry uncertainty parameters that perform well during navigation, the localization occasionally failed during loading. For this reason, during loading, the odometry linear noise parameter proportional to vehicle translation (odom_alpha3 as defined by AMCL) is increased. Additionally, once loading is complete, AMCL is reinitialized with a high covariance in order to obtain an appropriate localization, i.e., the convergence of the localization process. This makes it so that, as the vehicle drives back from the loading spot—at a fixed distance—AMCL converges to its true position, allowing the autonomous navigation to continue.

### 3.5. Incremental Map Update

Unlike sub-level stoping and block caving, in room and pillar type mines, the pile location continually moves as with each blast the tunnel is expanded. Therefore, in order to maintain autonomous operation, the map of the AOZ needs to be updated periodically. The same process as that described in Section 3.2 and Section 3.3 is performed, producing a map of the tunnel to be updated. Both these grid maps are produced by the GMapping package and therefore correspond to an image with the occupancy of each pixel, plus the resolution and origin (resolution, $O_{x}$, $O_{y}$), which allows one to transform pixel coordinates into world coordinates. The images produced by AMCL only have three pixel values, corresponding to occupied, free space, and unknown. Then, a heuristic process to merge the existing map *A* with the new update map *B* using OpenCV, outlined in Algorithm 1, is used. The morphologicalClose(·) function corresponds to a morphological close operation on the pixels with an occupied state. The denoise(·) function corresponds to removing connected components with less than three pixels with an occupied state. The align(GBlur(A.image),GBlur(B.image)) function corresponds to finding the transformation that registers B to A by maximizing the correlation between the walls with Gaussian blur [56], initializing the registration with the localization from the existing system at the start of the map update recording. This algorithm produces an updated map *C*.
**Algorithm 1:** Map-merging algorithm 
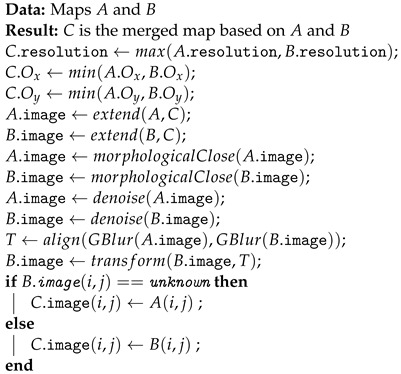


The procedure described in this section can be carried out online in the machine, or using recorded data. Requirements are that the LHD does not make sudden motions or go extremely fast. This can be accomplished by driving slowly in the area to be updated, or since the vehicle will be performing autonomous loading in that same area anyway, the data produced by the system while positioning prior to loading the pile can be used for this purpose.

## 4. Experimental Results: LHD Autonomous Operation in a Room and Pillar Salt Mine

### 4.1. Vehicle and Sensors

**Figure 6:**
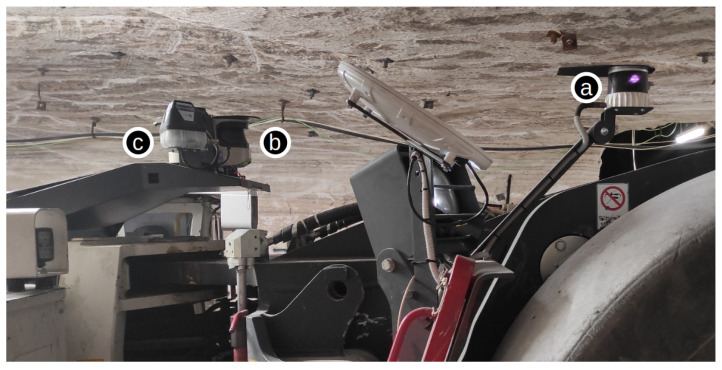
Picture of articulation section of LHD where LIDARs sensors are mounted: (a) 3D LIDAR; (b) 2D LIDAR directed towards bucket side; and (c) 2D LIDAR directed towards engine side.

Experimental results will be shown in an autonomous LHD, operating in a real room and pillar mine. The LHD vehicle, also referred to as front loader, is an articulated vehicle with hydraulic actuators capable of moving the main articulation as well as the bucket, which has two degrees of freedom—one for lifting its bucket and the other for rotating it. The LHD used in the tests is a GHH SLP-14H [2], whose dimensions are: length of 11,529 (mm) (in tramming position), width at drive unit of 3180 (mm), and height of 1883 (mm) above the canopy. Its operating weight is 482,000 (kg), while its payload is 14,000 (kg). A diagram showing an LHD with the positions of the sensors is shown in Figure 6, wherein the positions are equivalent to the ones in the simulated model from Figure 2.

Three LIDARs were mounted on the vehicle: one 3D LIDAR and two 2D LIDARs. The 3D LIDAR model is an Ouster OS2-32 below horizon [57], which provides a 22.5° vertical field-of-view with 32 layers and a 360° horizontal field-of-view. It was mounted on the bucket side of the articulation, specifically to measure and detect the ore piles that the vehicle has to load. For this same reason, the 3D LIDAR is slightly tilted downward in the frontal direction. The 2D LIDARs corresponds to the model Sick LMS511-10100 PRO [58], which provides a 190° horizontal field-of-view. One 2D LIDAR is mounted pointing forward, and the other is mounted pointing backward, covering 360° in total. Both 2D LIDARs are mounted on the rear half body, 800 (mm) above the 3D LIDAR. Communication between the autonomous driving computer and the machine’s programmable logic controller was handled through controller area network (CAN) protocol. This transmitted the internal variables of the machine to the computer, i.e., the wheel speed, engine RPM, articulation and bucket angles, hydraulic pressures, etc.

### 4.2. Experimental Conditions

**Figure 7:**
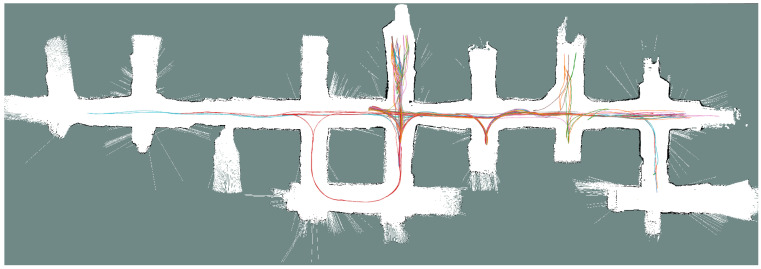
Trajectories during the final testing of the autonomous system, each mission trajectory is plotted in a different color.

The experimental validation of the whole autonomous LHD was carried out in the Werra Potash Mine, located in Philippsthal, Germany. The autonomous LHD system was pre-tested inside the mine’s AOZ during 10 months of continuous development of the autonomous loading and navigation system. The loading system was pre-evaluated in four different loading zones over these months. Once a loading zone was completely cleaned, a new blasting process was performed for continuing loading with the same zone, as occurs in a real mining production process. Through this process, the AOZ was expanded forward on each loading zone and the map of the area had to be updated, providing real testing opportunities for the map update process. This period ended with a final test of the autonomous loading and navigation system: during 2 days, the autonomous system performed a total of 26 missions, including navigation, navigation and loading, or navigation and dumping. Figure 7 shows the localization trajectories during all the tests on these 2 days. The parameters used for the experiments are shown in Table 3. Importantly, during loading and when the loading process results in the bucket being very full, the front-facing LIDAR sensor is partially blocked by the ore in the bucket, making the localization more challenging.

### 4.3. Mapping

**Figure 8:**
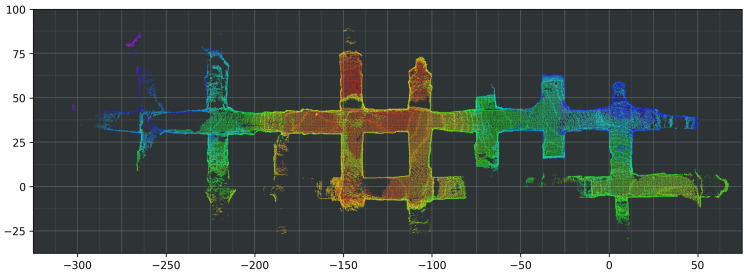
Three-dimensional mapping results using trajectory from 3D LIDAR-based LeGo-LOAM [20], top–down view. The 3D points are colored according to the height coordinate (*z* axis).

**Figure 9:**
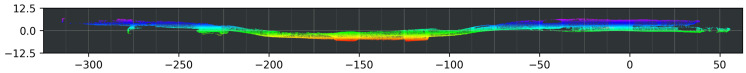
Three-dimensional mapping results using trajectory from 3D LIDAR-based LeGo-LOAM [20]; side view showing the elevation differences in mine tunnels. The 3D points are colored according to the height coordinate (*z* axis).

At the start of the pre-test period of the autonomous system, a professional LHD driver slowly drove the LHD (approximately 5 km/h) throughout the whole AOZ once, while sensor data were recorded. This process took 12 min. Applying LeGo-LOAM to the 3D LIDAR data produced an estimated 3D trajectory for the vehicle. Figure 8 and Figure 9 show the 3D LIDAR data overlaid using the estimated trajectory. In Figure 8, which shows the top–down view, the consistency of the overlaid LIDAR point clouds can be seen. From this, plus the fact that this visually matches the layout of the AOZ, it can be inferred that the trajectory estimate is very accurate. Figure 9 shows a side view of the same data, from which the elevation changes in the map can be seen, which also match the layout of the AOZ.

**Figure 10:**
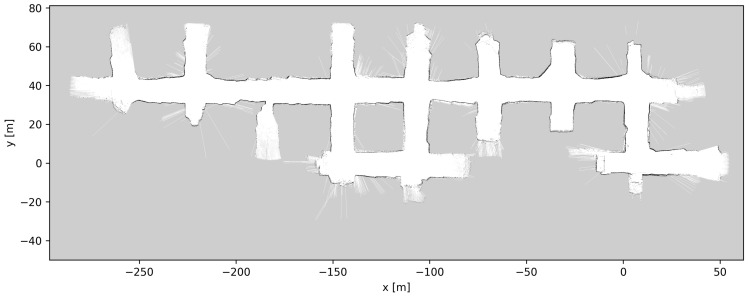
Two-dimensional mapping results using trajectory from 3D LIDAR-based LeGo-LOAM [20].

**Figure 11:**
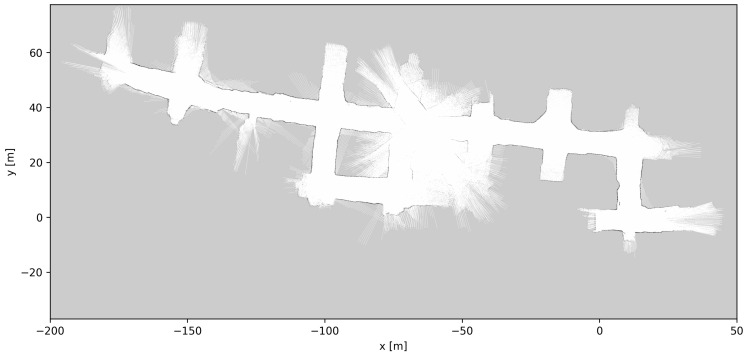
Mapping results using 2D LIDAR-based SLAM algorithm gmapping [15].

**Figure 12:**
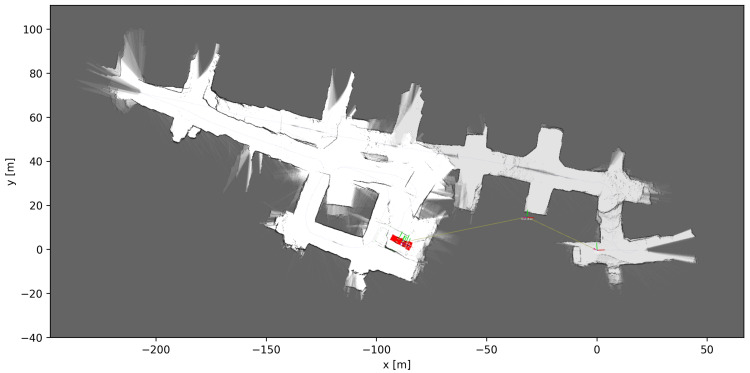
Mapping results using 2D LIDAR-based SLAM algorithm Google Cartographer [16], the difference in the shade of gray from Figure 10 and Figure 11 is due only to the use of the graphical interface.

Figure 10, Figure 11 and Figure 12 show the grid maps produced by the proposed solution, Google Cartographer and Gmapping, respectively. As can be seen from Figure 11 and Figure 12, both the algorithms which exclusively rely on 2D data produce unsatisfactory results. Google Cartographer produces an inconsistent map throughout, while Gmapping produces a good estimate until the trajectory makes a loop, at which point the rest of the mapping immediately diverges. These poor results can be explained due to the fact that the flat world assumption of the 2D SLAM algorithms is broken in the mine environment, as can be observed in Figure 9. Although these tunnel inclinations do not look major, in practice, the 2D LIDAR rays would frequently hit the ceiling and floor of the tunnel. And, even when hitting the tunnel walls, these were not perfectly vertical, making the flat world assumption even worse. On the contrary, the proposed algorithm produces a globally consistent 2D grid map of the entire AOZ.

### 4.4. Self-Localization

It should first be noted that, due to the inconsistent grid maps produced by the 2D LIDAR-based SLAM algorithms, testing the localization performance based on these mapping outputs does not make sense. Therefore, the localization performance is only shown with the proposed map-building methodology.

The localization system was in operation inside the mine’s AOZ during the 10-month pre-test period. During this period, the system worked satisfactorily while navigating. However, while testing the autonomous loading system, a problem with occasional delocalization was identified and a separate localization mode for loading was developed. During the 2 days of final testing, only a single delocalization event occurred. In this event, the autonomous navigation stopped itself due to an increased localization covariance. Localization was then remotely reinitialized and autonomous navigation resumed.

**Figure 13:**
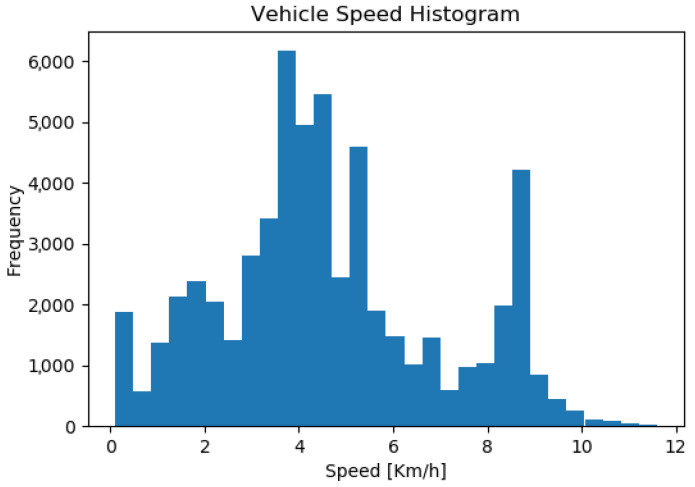
Speed histogram of the autonomous LHD during autonomous navigation. The total time for autonomous navigation over the 2 days of testing is 97 min.

Figure 13 shows a histogram of the speeds during autonomous navigation. During the 2 days of testing, the total autonomous navigation time amounted to 97 min. It can be noted that the distribution is bimodal around 4 (km/h) and 8.5 (km/h), which are the autonomous navigation speed targets during turning and when navigating a straight path, respectively.

**Figure 14:**
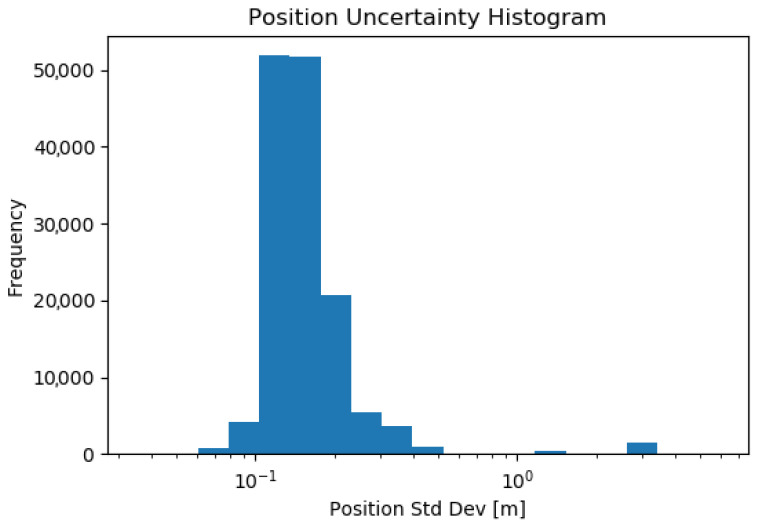
Histogram of the estimated position standard deviation given by AMCL, during all 26 tests. The total time of autonomous operation over the 2 days of testing is 236 min.

**Figure 15:**
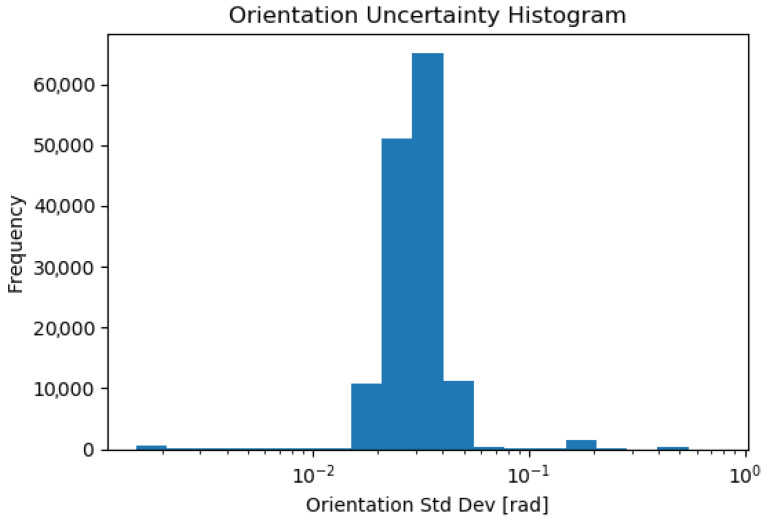
Histogram of the estimated orientation standard deviation given by AMCL during all 26 tests. The total time for autonomous operation over the 2 days of testing is 236 min.

Figure 14 and Figure 15 show histograms of the position and orientation standard deviations, extracted from the covariance reported by AMCL. It can be noted from these histograms that the uncertainty is occasionally much higher than during normal operation. This corresponds to the initialization of the localization pose.

**Figure 16:**
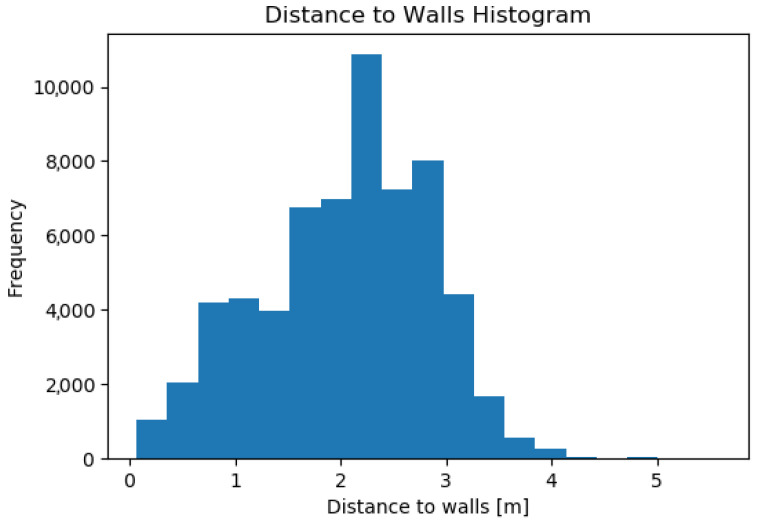
Histogram of the distance to walls during autonomous navigation during all 26 tests. The total time for autonomous navigation over the 2 days of testing is 97 min.

Figure 16 shows the distance to the walls during autonomous navigation as measured by the autonomous system using the 2D LIDAR. It should be noted more than 95% of the time, the navigation system is able to maintain a distance to walls higher than 60 cm.

**Figure 17:**
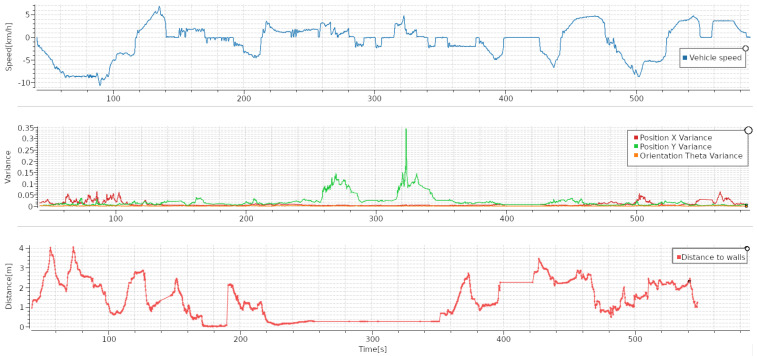
Speed in km/h (**top** graph), covariance (**middle** graph), and distance to walls (**bottom** graph) during navigation task 1. Route was straight along the main tunnel of AOZ. Each test trajectory is plotted in a different color.

**Figure 18:**
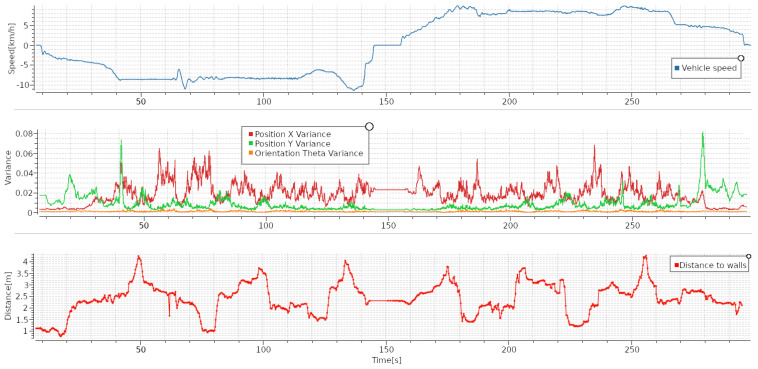
Speed in km/h (**top** graph), covariance (**middle** graph), and distance to walls (**bottom** graph) during navigation task 1. Route was straight along the main tunnel of AOZ.

As an example of the data collected, 2 out of the 26 tests are shown in Figure 17 and Figure 18. Figure 17 shows an example of a mission in which the machine navigates to the pile, loads some material, and then navigates to the dumping point; finally, it positions itself exactly where the material should be dumped. In this trial, the machine made two loading attempts, which can be observed in the position covariance. Since the Y covariance increases substantially when the LHD pushes against the ore pile (in the AOZ, the machine always loads ore in the Y direction).

Figure 18 shows an example mission where the machine drives along the main tunnel of the AOZ all the way to each end of the tunnel. It can be seen that the machine accelerates up to maximum speed and maintains it with some perturbations because of the changes in the inclination of the tunnel. In this test, we can see that the uncertainty is now higher in the X direction, which corresponds to the direction of the AOZ main tunnel, although it remains a full order of magnitude lower than the uncertainty while pushing against the pile. We conclude that, when navigating, the uncertainty is higher along the direction that the vehicle is moving.

### 4.5. Map Updates

**Figure 19:**
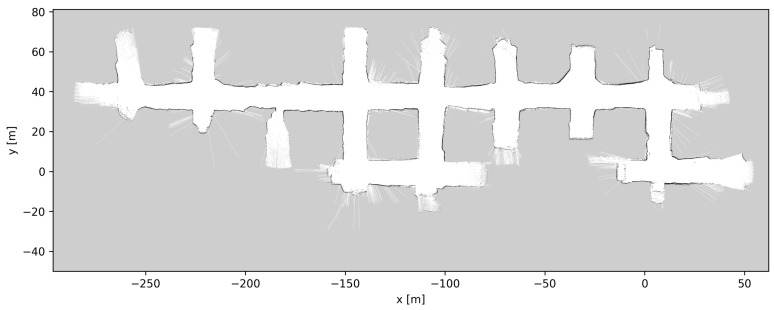
Initial map.

**Figure 20:**
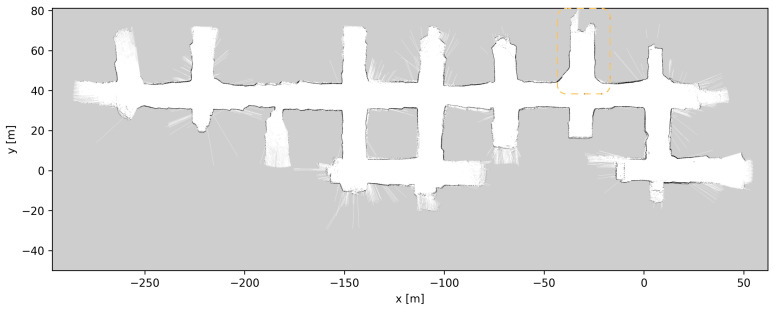
Map update steps: map after the first update, the updated section of the map is highlighted with a dashed orange line.

**Figure 21:**
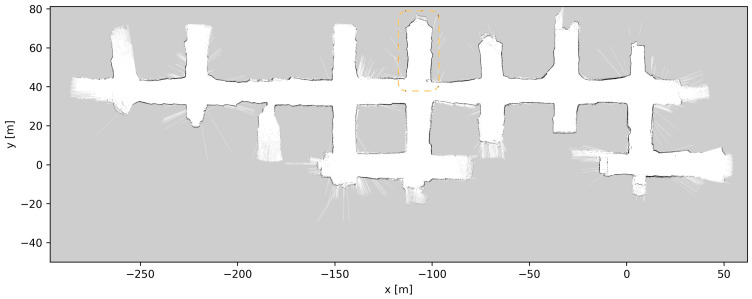
Map update steps: map after the second update, the updated section of the map is highlighted with a dashed orange line.

**Figure 22:**
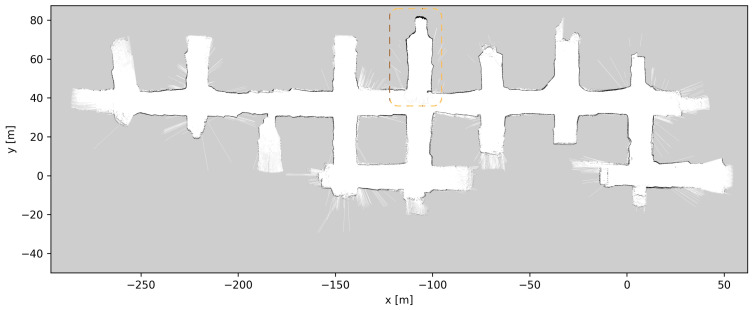
Map update steps: map after the third update, the updated section of the map is highlighted with a dashed orange line.

**Figure 23:**
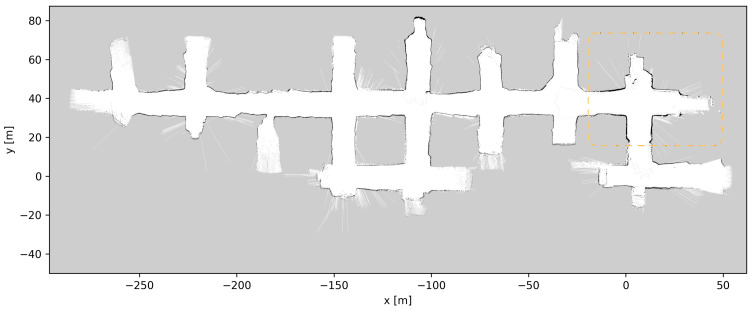
Map update steps: map after the fourth update, the updated section of the map is highlighted with a dashed orange line.

**Figure 24:**
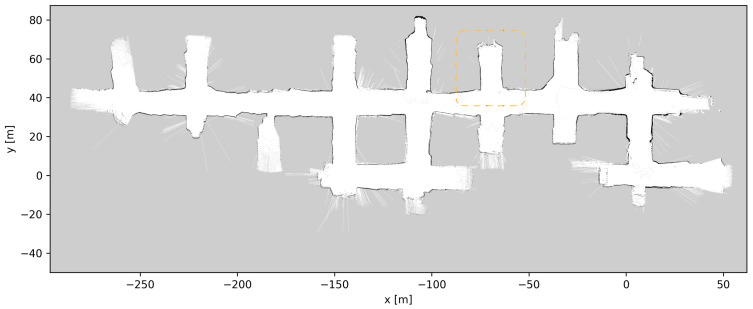
Map update steps: map after the fifth update, the updated section of the map is highlighted with a dashed orange line.

**Figure 25:**
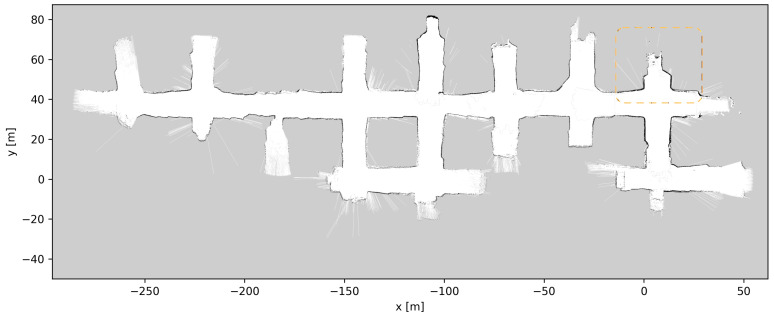
Map update steps: map after the sixth update, the updated section of the map is highlighted with a dashed orange line.

Throughout the development and testing of the system, the blasts at the different loading tunnels extended each tunnel length. After each blasting and loading of the produced ore, the update procedure described in Section 3.5 was performed, a total of six times. Figure 19, Figure 20, Figure 21, Figure 22, Figure 23, Figure 24 and Figure 25 show the map at each step of the update process. It can be seen that, at each update, one tunnel extends a bit, while the rest of the map stays the same. In each figure, the updated section of the map is highlighted with a dashed orange line. In Figure 20, the tunnel is extended near (x,y)=(−30,60), after a blast. Figure 21 extends the tunnel near (x,y)=(−110,60), also after a blast. Figure 22, Figure 23, Figure 24 and Figure 25 extend the tunnel near (x,y)=(−110,80), (x,y)=(0,45), (x,y)=(−60,60), and (x,y)=(0,45), respectively. Note that steps shown in Figure 22 and Figure 25 do not correspond to new blasts but to more of the newly blasted tunnel being exposed after repeated loads of material being removed. It should be noted that the final testing shown in the previous section was all performed with the final updated map from Figure 25. However, the other maps were all used during the months of testing without issues. After many map updates, a complete remapping of the AOZ could be applied to eliminate any inconsistencies introduced by the heuristic nature of the map merging process. Given that the initial mapping was performed in 12 min, this would not introduce major production issues.

## 5. Discussion

In this article, a methodology to use 2D and 3D SLAM algorithms to produce accurate 2D grid maps was introduced. In contrast with state-of-the-art pure 2D SLAM solutions, which were not able to produce a consistent 2D grid map due to the non-planar geometry of the room and pillar mine testing environment, the proposed methodology was able to produce accurate and consistent 2D grid maps, allowing for robust 2D localization in a real room and pillar mining environment.

Using the superior grid map produced by this procedure, autonomous loading and navigation tests were performed, in which AMCL-based localization was able to maintain the LHD’s localization within bounds, with only one delocalization event in 26 tests. This event was detected by measuring the reported uncertainty covariance, and localization was reinitialized remotely.

The proposed methodology can be used to localize a vehicle in tunnels that are only roughly 2D while using only 2D LIDARs during localization. Only initial mapping and map updates require a 3D LIDAR; therefore, if automating a fleet of vehicles, only a single vehicle could be equipped with a 3D LIDAR. However, if operating in a slowly changing environment, the 3D sensor could only be mounted during the initial setup and periodic map updates, thus keeping the expensive sensor safe from the harsh environment that is an operating mine. Given that the localization only occurs in 2D using the 2D LIDAR sensor, the computational cost of the localization is very low, leaving the computation time available for the other autonomous processes that have to run onboard the LHD.

Another advantage of the proposed system is the short initial setup/commissioning of the system. The mapping of the environment only requires a single drive through the environment with the 3D LIDAR and the SLAM/map-building algorithms being able to be run in real-time. In the tests performed in a real room and pillar mine, the data collection for map building took 12 min. The produced map can then immediately be used, although a human check of the consistency of the grid map before the start of operation is recommended. However, due to the fact that the system was in development at the time, the initial map building was not run in real time. This was developed later and the system was capable of running in real-time, just like the map update process.

Limitations of this work include the case in which a tunnel goes directly over the top or underneath another tunnel; in this case, the pure 2D localization is not able to differentiate between the tunnels. This limitation could be overcome without full 3D mapping by combining the topological and 2D metric maps, dividing the area into metric 2D sub-maps. Another limitation of this approach is the performance of the underlying 3D map-building algorithm. This can either be overcome by replacing the 3D SLAM algorithm as the state-of-the-art progresses, or similarly to before, by making a topological representation using metric sub-maps in which each metric sub-map size can be limited by the performance of the 3D LIDAR-based SLAM algorithm being used.

Future work on localization includes autonomous re-localization in the case of a delocalization event, as well as the use of the 3D SLAM system to produce 3D models of the piles to be loaded.

## Figures and Tables

**Table 1:** Approaches to LIDAR-based mine map building. Examples of existing state-of-the-art algorithms corresponding to each approach are shown in the table cells.

	3D Sensor	2D Sensor
3D map	LOAM, LeGo-LOAM	vertical + horizontal, 2D LIDAR
2D map	*Proposed solution *	Cartographer, Gmapping

**Table 2:** Approaches to LIDAR-based localization. Examples of existing state-of-the-art algorithms corresponding to each approach are shown in the table. Approaches to 3D localization with a 2D map are not known to the authors, and therefore, this cell is greyed out.

	3D Localization	2D Localization
3D map	DLL, NDT-3D, AMCL3D	NDT-2D, AMCL
2D map	*not possible *	AMCL, NDT-2D

**Table 3:** Parameters used during autonomous operation tests.

System	Parameter	Value
AMCL	laser_min_range	0.1 m
AMCL	laser_max_range	35 m
AMCL	laser_max_beams	360
AMCL	laser_z_hit	0.95
AMCL	laser_z_short	0.1 m
AMCL	laser_z_max	0.05 m
AMCL	laser_z_rand	0.1 m
AMCL	laser_sigma_hit	0.1 m
AMCL	laser_lambda_short	0.05 m
AMCL	odom_alpha1	0.08
AMCL	odom_alpha2	0.05
AMCL	odom_alpha3	0.05
AMCL	loading_odom_alpha3	0.09
AMCL	odom_alpha4	0.08
AMCL	odom_alpha5	0.05
AMCL	min_particles	300
AMCL	max_particles	10,000
AMCL	kld_err	0.05
AMCL	kld_z	0.99
Pullback	Pullback distance	3 m

## Data Availability

The data presented in this study are contained in the article itself.
